# Integrating genome-wide and epigenome-wide associations for antipsychotic induced extrapyramidal side effects

**DOI:** 10.1007/s00213-025-06912-w

**Published:** 2025-10-11

**Authors:** Kai Yao, Johan H. Thygesen, Siobhan K. Lock, Antonio F. Pardiñas, Antonia L. Pritchard, Israa Elbashir, Abdullah Lodhi, Zain-Ul-Abideen Ahmad, Michael C. O’Donovan, Michael J. Owen, James T. R. Walters, David St Clair, Nick Bass, Andrew McQuillin

**Affiliations:** 1 Molecular Psychiatry Laboratory, Division of Psychiatry, University College London, Rockefeller Building, 21 University Street, London, WC1E 6BT UK; 2 Institute of Health Informatics, University College London, 222 Euston Road, NW1 2DA London, UK; 3 Centre for Neuropsychiatric Genetics and Genomics, School of Medicine, Cardiff University, Hadyn Ellis Building, Cardiff, CF24 4HQ Cardiff, UK; 4 Division of Biomedical Science, University of the Highlands and Islands, An Lochran, Inverness, IV2 5NA UK; 5 School of Medicine, Medical Sciences, Nutrition University of Aberdeen, King’s College, Aberdeen, AB24 3FX UK

**Keywords:** Genome-wide association, Epigenome-wide association, Schizophrenia, Antipsychotic, Extrapyramidal side-effects

## Abstract

**Rationale:**

Antipsychotic medications are the first-line treatment for schizophrenia. Around 40% of people who are treated with antipsychotics could develop extrapyramidal side-effects (EPSE) including: (1) Dyskinesia, (2) Parkinsonism, (3) Akathisia, and (4) Dystonia.

**Objectives:**

This study aimed to identify genetic risk factors for EPSE presence following antipsychotic treatment.

**Methods:**

We conducted Genome-wide association (GWAS) and Epigenome-wide association (EWAS) meta-analyses of EPSE, with subset analyses separating first and second generation antipsychotic (FGA/SGA) exposure. We integrated significant EWAS findings from a between-case design to a comparable GWAS for association enrichment. We investigated whether polygenic risk scores (PRS) for schizophrenia, Parkinson’s disease, and Lewy-body dementia could predict EPSE.

**Results:**

The primary GWAS top SNP rs2709733 (A/G) (*p* = 5.755 × 10^−07^) mapped to a long intergenic non-protein coding RNA, *LINC01162* with consistent effects across all cohorts. Subset analyses with distinct FGA exposure indicated suggestive genes such as *NAV2*, *NRG3*,* LSAMP* and SGA exposure indicated *SHISA9* and *CNBD1* which are relevant for schizophrenia, autism, and epilepsy. In our primary EWAS, the most significant differentially methylated position (DMP) was cg05599348 (3.181 × 10^−07^), located at chrX:103,174,718 (hg19) mapping to *TMSB15B*. Comparing EPSE cases to healthy controls, we identified nine DMPs associated with EPSE. The DMP cg12044923 (chr2:241453995, hg19), located within the *STK32B* gene, showed significant enrichment for EPSE association (permutation *p* = 0.010). *STK32B* is relevant to both psychiatric and movement disorders, suggesting potential shared mechanisms.

**Conclusions:**

Our study sheds new light on the potential biological mechanisms underlying EPSE development in schizophrenia, highlighting the importance of exploring both methylation changes and SNP associations.

**Supplementary Information:**

The online version contains supplementary material available at 10.1007/s00213-025-06912-w.

## Introduction

Antipsychotic medications are the first-line treatment for schizophrenia (Sabe et al. 2022). Although many people benefit, around 70% may experience treatment failure such as psychiatric rehospitalization, suicide attempt, discontinuation or switch to other medication (Tiihonen et al. 2017). Extrapyramidal side-effects (EPSE) are common with antipsychotic treatment (Carbon et al. 2018; Huhn et al. 2019), with approximately 40% of patients treated with first-generation antipsychotics (FGA) experiencing EPSEs (Wubeshet et al. 2019). EPSEs still occur with second-generation antipsychotics (SGA), although at lower rates in comparison with FGA (Divac et al. 2014). FGAs are primarily dopamine D2 receptor antagonists, which reduce dopaminergic activity to alleviate positive symptoms of psychosis. This, however, often leads to motor side effects such as EPSE (Kapur and Remington 2001). Meanwhile, SGA targets both dopamine D2 and other receptors such as serotonin *5-HT2A* receptors. Serotonin modulation offsets some dopamine blockade effects thus reducing EPSE (Leucht et al. 2009; Zhang et al. 2013). EPSEs describe the movement abnormalities induced by antipsychotics including:

1. *Dyskinesia*, hyperkinetic choreiform involuntary movements of the face, extremities, and the trunk (Lim et al. 2021). When these abnormal movements emerge after at least a few months of antipsychotic exposure and persist for more than one month, this condition is termed tardive dyskinesia (TD). TD can sometimes become chronic and is often distinguished from acute dyskinesia by its delayed onset and persistence.
2. Parkinsonism, symptoms of rigidity, tremor and impaired or slow movement (bradykinesia) (Keener, and Bordelon, 2016).
3. Akathisia, characterised by subjective inner restlessness and objective increase in motor activity such as pacing (Factor, et al., 2008).
4. Dystonia, characterised by sustained and abnormal contractions, that can result in abnormal movements and postures (Harten, and Kahn, 1999).

These movement abnormalities can lead to severe impairment and reduction in the quality of life of individuals with schizophrenia (D’Souza and Hooten 2023), by interfering with daily living activities and social functioning (Schouten et al. 2012; Fujimaki et al. 2012). In a meta-analysis, the prevalence of spontaneous dyskinesias and parkinsonism was found to be higher in antipsychotic-naive patients with schizophrenia and in first-degree relatives of patients with schizophrenia as compared to healthy controls, indicating a heritable, non-drug induced component to these abnormalities (Koning et al. 2010).

Parkinsonism seen in EPSE can be clinically indistinguishable from the movement abnormalities seen in the neurological disorders like Parkinson’s disease (PD) and Lewy Body Dementia (LBD). Previous studies have identified shared significant loci between schizophrenia and PD (Nalls et al. 2019; Smeland et al. 2021). For example, schizophrenia and PD are both associated with the 22q11.2 deletion syndrome (Jonas et al. 2014). A duplication of the *SNCA* gene, for which pathogenic variants are associated with autosomal dominant Parkinson’s and encodes α-synuclein, a major constituent of LBD, was reported in an individual diagnosed with schizophrenia nine years prior to the development of mild Parkinsonism (Takamura et al. 2016). A recent neuroimaging study on individuals with first episode psychosis found that higher iron loading in the basal ganglia correlated with greater motor abnormalities including EPSE (Cuesta et al. 2021). Similar associations were found with motor abnormalities in PD (Ward et al. 2014; Kim and Wessling-Resnick 2014). In view of this, it is plausible that there are shared genetic features between these disorders which also contribute to the shared phenotypical features including movement abnormalities like EPSE in schizophrenia.

Genome-wide Association Studies (GWAS) are a promising approach to identify potential genes associated with development of EPSE given the often-complex biological pathways implicated in psychiatric traits (Duncan et al. 2019). However, to our knowledge, only one past study investigated antipsychotic induced EPSE using GWAS comparing EPSE cases versus EPSE controls among European schizophrenia samples (Åberg et al. 2010). The genotype data in that study had somewhat limited genomic coverage compared to contemporary studies and furthermore there was no imputation of genotypes not captured on the genotyping array. Other studies have examined EPSE presence by comparing EPSE cases with healthy controls (Levchenko et al. 2021) and by analysing mixed ancestry cohorts (Lim et al. 2021). Epigenome-wide Association Study (EWAS) allows for the examination of environmentally induced methylome variation which could directly result from chronic antipsychotic exposure (Wagner et al. 2014; Murphy and Mill 2014). To date, there has been no EWAS on EPSE to examine the influence from antipsychotics.

Our understanding of the molecular mechanisms underlying EPSE may be improved using an integrated functional genomics strategy. The overall aim of this study was to conduct an integrated GWAS and EWAS meta-analysis of EPSE data from existing schizophrenia studies. We also investigated whether Polygenic Risk Scores (PRS) for schizophrenia, PD and LBD could be used to predict the risk of the development of EPSEs. The findings could provide a better understanding of the genetic underpinnings of EPSE and pave the way for the identification of informative genetic biomarkers that could allow for specific tailoring of treatments in the future.

## Method

### Participant selection and genotyping

#### UCL Participants

All UCL participants received an ICD10 diagnosis of schizophrenia from a UK National Health Service (NHS) psychiatrist. Details have been reported elsewhere (World Health Organization 1992; Trubetskoy et al. 2022). Semi-structed interviews were performed to collect participants’ basic and medical information following Schizophrenia and Affective Disorder Schedule (SADS-L) (Spitzer et al. 1978) and the 90-item Operational Criteria Checklist (OPCRIT) (McGuffin et al. 1991). Ancestrally matched healthy controls were recruited from the National Health Service (NHS) blood transfusion service and from study sites where case participants were also being recruited. The healthy controls were screened for an absence of a lifetime history of the following disorders: schizophrenia and any other psychosis, major affective or schizoaffective disorders, eating disorders, alcohol/drug addiction, and obsessive-compulsive disorders. All participants read an approved information sheet and signed a physical informed consent form. The study was approved by the NHS Metropolitan Multi-centre Research Ethics Committee (MREC/03/11/090). Genome-wide single nucleotide polymorphism data were generated in three waves at the Broad Institute, Boston, MA, US, using the, Affymetrix Array, Illumina PsychArray, and Illumina Global Screening Array (GSA). The three waves of data underwent equivalent quality control and imputation methods which had been described in details elsewhere (Grigoroiu-Serbanescu et al. 2020).

#### Aberdeen Participants

The Aberdeen case–control sample has been described elsewhere (Stone et al. 2008). Briefly, the cohort contains participants with schizophrenia and healthy controls who have self-identified as born in the British Isles (95% in Scotland). All participants with schizophrenia met the Diagnostic and Statistical Manual for Mental Disorders fourth edition (DSM-IV) (American Psychiatric Association 1994) and ICD-10 criteria for schizophrenia (World Health Organization 1992). Controls were volunteers recruited through general practices in Scotland. Volunteers who replied to a written invitation were interviewed using a short questionnaire to exclude major mental illness in the individual themselves and their first-degree relatives. The study was approved by both local and multiregional academic ethical committees and all cases and controls gave informed consent. The samples were genotyped at the Broad Institute, as described for the UCL participants.

#### Cardiff Participants

Were recruited from community mental health teams in Wales and England on the basis of a clinical diagnosis of schizophrenia or schizoaffective disorder (depressed sub-type) as described previously (Carroll et al. 2011). Diagnosis was confirmed following a SCAN interview (Wing et al. 1990) and review of case notes followed by consensus diagnosis according to DSM-IV criteria (American Psychiatric Association 1994). The UK Multicentre Research Ethics Committee (MREC) approved the study and all participants provided informed consent. The samples were genotyped at the Broad Institute, as described for the UCL participants.

#### UK Biobank (UKB) Participants

UKB is a biomedical database and research resource of approximately 500,000 individuals from across the UK aged 40 to 69 years at recruitment (between 2006 and 2010) (Sudlow et al. 2015). Potential participants in UKB were selected using diagnosis of schizophrenia from ICD10, including codes from F20.0 to F20.9 and excluding participants with any primary Parkinson disorder with G20.

### Coding of extrapyramidal side-effects data

EPSE coding was based on data obtained through clinical interviews and medical records from existing studies, as described above for each sample. The record may only captured treatment history up to the time of the clinical interview and blood draw. The EPSE coding followed: (1) prescription of antipsychotic medications (FGA or SGA); (2) recorded clinical features of EPSE; and/or recorded medications prescribed to alleviate EPSE. We used key terms to classify participants with schizophrenia as cases (having EPSE) or controls (not having EPSE). These key terms covered two main areas, behavioural and pharmacological (Supplementary Tables 1 and 2):

1. *Behavioural features of the four types of EPSE (dystonia*,* akathisia*,* parkinsonism*,* and dyskinesia)* To compile a list of keywords for each of these EPSE types, we consulted several rating scales that are frequently employed to measure EPSE including: The Abnormal Involuntary Movement Scale (AIMS) (Munetz and Benjamin 1988), the Extrapyramidal Symptom Rating Scale (ESRS) (Chouinard and Margolese 2005), The Simpson Angus Scale (Hawley et al. 2003), and the Barnes Akathisia Rating Scale (BARS) (Barnes 1989). In addition, we searched reliable sources of clinical information for each of these abnormalities including the National Institute for Health and Care Excellence (NICE) guidelines (NICE 2014) and the BMJ Best Practice (BMJ Best Practice 2021).
2. *Pharmacological treatments for EPSE* To generate key words for pharmacological treatments for EPSE, we searched The NICE guidelines (NICE 2014) and The Maudsley Prescribing Guidelines in Psychiatry (Taylor et al. 2021) for the most recent recommendations on managing EPSE to identify a list of medications.

The UCL and Aberdeen participants’ EPSE status was derived using the same list of key words described in Supplementary Tables 1 and 2. The Cardiff participants’ EPSE status coding had a few minor adaptions. The keywords “dribbling” was added as it better captured other saliva-related key-words; ‘shakes’ was removed as it was described in the context of anxiety; “still” was removed as it referred to still doing something not being physically still; “tap” was removed as it was in the context of ‘tapered’; “march” was removed as it referenced the month of March; “irritable” was removed as it was in the context of IBS/irritable bowel syndrome; “parkin” was removed as it referred to Parkinson’s disease not parkinsonism; ‘tropin’ was excluded as it captured atropine as opposed to benzatropine. The UKB participants were retained if they received any FGA or SGA (medication codes in Supplementary Tables 3 and 4), then stratified by whether participants received any medication to treat EPSE (EPSE medication codes in Supplementary Table 5); diagnosis of other drug-induced secondary Parkinsonism in G21.1; Drug-induced dystonia in G24.0 or Drug-induced tremor in G25.1.

To enable subset analyses with different FGA and SGA exposure, the overall EPSE coding was further split into two groups based on the participants’ medication history. The participants either had any exposure to FGA or only exposure to SGA. We stratified the antipsychotic exposure into separate groups to compare their differential effects, given FGAs’ higher EPSE prevalence and distinct mechanistic profiles.

### GWAS meta-analyses and follow-up analyses

For the main analysis, we took a within case design comparing participants with exposure to FGA or SGA with EPSE vs. not having EPSE. We also conducted subset analyses separating participants who had any exposure to FGA (including those also exposed to SGA) and participants only exposed to SGA. The Cardiff samples were only included in the SGA subset given most participants only had SGA exposure.

We applied logistic regressions taking the participants’ EPSE status to evaluate the association between imputed SNP dosages. For UCL participants, we performed separate GWAS for data from each wave using PLINK v2.00a2LM (Purcell et al. 2007). We conducted the same sets of analyses for Aberdeen, Cardiff, and UKB samples separately. The participants’ age, sex and the first ten principal components of population structure were included as covariates to control for population stratification.

We conducted fixed-effect meta-analysis taking each GWAS’s effective sample sizes (Neff) as weights using METAL (see calculation of Neff in Supplementary Table 6) (Willer et al. 2010). We also conducted the METAL Heterogeneity analysis to test of the observed effect sizes were homozygous across samples. The Genome-wide significance threshold was set at 5× 10^−08^ to account for approximately one million independent variants tested. The output results were uploaded to FUMA for interpretation (Watanabe et al. 2017). We also conducted a binomial sign test to evaluate the SNP associations between the FGA and SGA subsets at 10^−03^ level. If there were no SNPs associations between the FGA and SGA subsets, the expectation is that less than 50% of the Z scores from the meta-analyses would be in the same direction.

### EWAS methylation data

Methylation data was only available for a proportion of the UCL and Aberdeen samples. The EZ-96 DNA Methylation kit (Zymo Research, CA, USA) was used to treat 500ng of DNA from each sample with sodium bisulfite in duplicate. DNA methylation was quantified using the Illumina Infinium HumanMethylation450 BeadChip (Illumina Inc.) run on an Illumina iScan System (Illumina) using the manufacturers’ standard protocol. Detailed data collection and imputation process has been described elsewhere (Hannon et al. 2016). As smoking status information was not present for all samples, we estimated a proxy based on the DNA methylation profile at sites known to be associated with smoking status following a previously described approach (Elliott et al. 2014). Cell composition data were not available for these DNA samples, therefore these were estimated Houseman algorithm (Houseman et al. 2012; Koestler et al. 2013) for seven variables recommended in the documentation. We also estimated the participants’ methylation age using the Epigenetic Clock software (Horvath 2013).

### EWAS meta-analyses

We employed the same design as in the GWAS to analyse the association of EPSE status on DNA methylation profiles. This included comparisons of EPSE presence among participants with any FGA/SGA exposure (111 EPSE cases, 203 EPSE controls), any FGA exposure (87 EPSE cases, 87 EPSE controls), and only SGA exposure (17 EPSE cases, 106 EPSE controls). DNA methylation values for each probe were regressed with covariates for methylation age, gender, seven cell composition scores, and smoking score. Then the results from UCL and Aberdeen were combined with fixed-effect meta- analyses.

These within-case analyses may be limited by sample size constraints, potentially reducing statistical power to detect subtle methylation changes. We also performed EPSE case with any antipsychotic exposure vs. healthy control analyses to boost statistical power (111 EPSE cases, 748 Healthy controls; sample demographics in Supplementary Table 8). To eliminate the influence of schizophrenia in the case control study design, we included participants’ schizophrenia PRS as an additional covariate. Thus, the EPSE case control design may Help reveal EPSE-associated methylation Changes attributable to long-term antipsychotic exposure after accounting for potential schizophrenia risks. The EWAS meta-analysis significance threshold was set at 1× 10^−07^ which is less stringent than the GWAS threshold, reflecting fewer tested CpG sites and higher correlation among methylation markers.

### EWAS findings integration and permutation test

We performed separate GWAS on the same participants used in the case control design EWAS following the same procedure described. The results were combined using METAL and clumped to represent LD independent loci in lead using the 1000 genome European samples as a reference (1000 Genomes Project Consortium et al. 2015). Any significant CpG sites from the EWAS were mapped to within 250 kb of each in the associated GWAS results to identify an enrichment in the region. To quantify significance, 5000 random permutations were generated. Empirical P values for each region were calculated by counting how many of the permutations had more significant P values than the mapped P value from GWAS and dividing by the total number of permutations performed. The CpG sites’ locations were also mapped to clumped schizophrenia GWAS results within 250 kb for comparisons (Trubetskoy et al. 2022). Regional plots were produced using GWASLab (He et al. 2023).

### PRS calculation and meta-analyses

We calculated the participants’ PRSs for schizophrenia, Lewy body dementia and Parkinson’s disease using the PRS-CS method with the latest available reference GWAS (Ge et al. 2019). We chose the European samples from the 1000 Genomes Project Consortium as our LD reference panel given all samples included were of European Ancestry (1000 Genomes Project Consortium et al. 2015). Once weights were produced, individual PRSs were calculated using PLINK v2.00a2LM (Chang et al. 2015). We then used the mean and standard deviation of the healthy controls’ PRSs from each sample to standardize their cases’ PRSs. The schizophrenia GWAS came from Trubetskoy et al. (PGC wave 3), which were derived exclusively from European samples (Trubetskoy et al. 2022). The GWAS statistics for PD came from European samples of Nalls et al. excluding 23andMe data (Nalls et al. 2019). The GWAS statistics for LBD came from Chia et al., only including European samples (Chia et al. 2021). We adapted the schizophrenia GWAS to exclude each sample’s participants used in the current study to avoid sample overlap. The new GWAS generation followed the same procedures as previously described (Trubetskoy et al. 2022).

We performed multiple logistic regression analyses to assess how these various PRSs predict the presence of EPSE in each sample among those who had any FGA/SGA exposure. Then the results were meta-analysed using a fixed effect model. The assumptions for logistic regressions were pre-checked and found to be satisfactory for each regression. The significant threshold was kept as 0.0167 (i.e. 0.05/3), for multiple testing correction.

## Results

### GWAS sample demographics

Overall, the main GWAS meta-analysis included 2471 participants with schizophrenia, of whom 1178 (48%) had EPSE. The participants had a mean age of 46.57 (SD 12.22) years old and were mostly males (70%; Table 1) as is typical of genomic studies of schizophrenia. All participants had antipsychotic exposure and most of the participants had taken at least one type of SGA (59%). The participants with and without EPSE did not differ in terms of age at assessment (46.53 vs. 46.62, *p* = 0.855) nor sex (males 71% vs. 69%, *p* = 0.213). EPSE was more prevalent in those who had taken FGA or were on both (FGA 51%, SGA 41%, both 61%; *p* < 0.001).

**Table 1:** GWAS participants’ demographics and clinical characteristics concerning EPSE presence

	*N*	Overall	EPSE Presence	EPSE Absence	*p*-values
Meta-Analyses	*2471*		*n= 1178 (48%)*	*n= 1293 (52%)*	
Age at assessment	2471	46.57 (12.22)	46.53 (11.82)	46.62 (12.59)	0.855^a^
Sex	2471				
Male		1727 (70%)	838 (71%)	889 (69%)	0.213^b^
Female		744 (30%)	340 (29%)	404 (31%)	
Antipsychotics	2425				
First Generation		488 (20%)	249 (22%)	239 (19%)	**< 0.001** ^b^
Second Generation		1436 (59%)	595 (52%)	841 (66%)	
Both Generations		501 (21%)	305 (26%)	196 (15%)	

*Notes.* *EPSE* extrapyramidal side effects, *SD* standard deviation

^a^ Two Simple t-test, mean (SD)

^b^ Pearson’s Chi-squared test of independence, n (%)

In bold p passed significance threshold

The participants’ characteristics differed between sample sets (Supplementary Table 6). The participants who developed EPSE were at an older age at assessment than those who did not in the UCL (46.33 vs. 42.72, *p* < 0.001) and UKB samples (56.06 vs. 53.94, *p* = 0.020; Supplementary Table 6). In the Cardiff sample, participants who developed EPSE had an earlier age of schizophrenia onset (24.30 vs. 27.50, *p* = 0.006). The pattern of EPSE being more prevalent in those who have taken FGA were consistent across most cohorts except for the Cardiff samples where most participants only had exposure to SGA (Supplementary Table 6). The varying prevalence of EPSE across different datasets may indicate that the format or detail of medical records can impact the sensitivity of EPSE detection.

For EPSE coding composite in the UCL sample as an illustration, the majority of EPSE cases were identified as having parkinsonism symptoms (327 cases, 58%), with small variations between FGA (56%) and SGA (63%) exposure subgroups (see details in Supplementary Table 7). Additionally, about 30% of cases had more than one type of EPSE features, with slightly higher percentages in the FGA (35%) than the SGA subgroup (25%).

### GWAS results

For the primary meta-analysis including all participants, we did not observe any SNP passing the Genome-wide significance threshold at 5× 10^−08^ (Table 2 and Supplementary Fig. 1). We found no evidence for population inflation across the samples given the lambda value of 1, suggesting the test statistics are not inflated by population stratification or cryptic relatedness (Supplementary Fig. 2). We observed no evidence for excessive heterogeneity across the samples. The top index SNP rs2709733 (A/G; Z = 4.999; *p* = 5.755 × 10^−07^) mapped to a long intergenic non-protein coding RNA, *LINC01162* and its effect was consistent across all cohorts (Table 2). The other affiliated protein-coding genes from the suggestive SNP rs11077391 (*p* = 3.765 × 10^−06^) included *USP36* and *CYTH1*.

**Table 2:** Regions of the genome showing the strongest association signals with EPSE presence for separate FGA and SGA exposure

Index SNP	Chr	Position	A1/A2	EAF	Z Score	*P*-values	*P* Het	Match	Weight	Mapped Genes
EPSEs with exposure to any antipsychotic										
rs2709733	7	20,878,995–20,955,370	A/G	0.452	4.999	5.755 × 10^−07^	0.268	++++++	1937	*LINC01162*
rs12662039	6	99,546,454–99,598,593	C/G	0.057	4.948	7.492 × 10^−07^	0.461	+++++?	1561	
rs11077391	17	76,661,207–76,789,754	A/G	0.311	4.624	3.765 × 10^−06^	0.952	+++++?	1561	*USP36*,* CYTH1*
rs62530097	9	7,590,958–7,667,847	T/G	0.090	4.494	6.978 × 10^−06^	0.560	++++++	1937	
EPSEs with exposure to any type of FGA*										
rs2028609	11	19,918,741–19,933,123	T/C	0.494	−5.144	2.693 × 10^−07^	0.955	--?--	660	*NAV2*
rs1416851	10	84,329,093–84,380,431	T/C	0.233	−4.927	8.365 × 10^−07^	0.290	-----	780	*NRG3*
rs17723244	3	117,509,984–117,822,025	A/G	0.721	−4.751	2.022 × 10^−06^	0.595	-----	780	*LSAMP*,* LINC03051*
rs2840001	3	168,714,097–168,862,366	A/G	0.275	4.725	2.306 × 10^−06^	0.563	+++++	780	
EPSEs with exposure to SGA only										
rs72800384	10	54,799,931–54,839,608	T/C	0.244	4.567	4.937 × 10^−06^	0.179	-+++++	1063	
rs117545352	8	87,786,629–87,894,786	A/G	0.933	−4.522	6.117 × 10^−06^	0.272	----+-	1063	*CNBD1*
rs4781355	16	13,021,889–13,101,555	A/G	0.637	4.509	6.524 × 10^−06^	0.776	++++++	1063	*SHISA9*

*Notes.* *FGA* first generation antipsychotics, *SGA* second generation antipsychotics. Index *SNP* the single-nucleotide polymorphism with the strongest association in the genomic region and each is independent at r^2^ < 0.1, *Chr* chromosome, Position the start and end position (UCSC hg19) of the SNP locus where near-by SNPs were clumped to with nominal associations (*p* < 0.05) and LD (r^2^ < 0.1) within 250-kb windows taking the 1000 Genomes project phase 3 EUR as LD reference; A1/A2, effect and alternate allele; *EAF* the effect allele frequency based on 1000 genomes EUR; Z-score, the meta-analysis output reference score for the SNP; P-values, the corresponding p-values to the candidate SNP; P Het, corresponding p-values to the degree of variability in effect sizes from METAL analysis; Match, the agreement across the six datasets, + means individuals who carry the A1 allele have positive EPSE association, - means negative,? means missing, the orders are: three sets of GWAS from UCL samples (1) Affymetrix Array, (2) Illumina PsychArray, (3) GSA, Illumina Global Screening Array, (4) Aberdeen samples, (5) UKB samples, and (6) Cardiff samples, Cardiff samples were excluded in the second subset (any FGA exposure) given most samples only had exposure to SGA; Weight, the overall Neff of the sample for the SNP; Mapped Genes, the top Genes mapped by positional mapping criterion with maximum distance 10 kb to the locus position. No SNP passed Genome-wide significant threshold at 5× 10^−08^.

*Includes participants who had also been exposed to SGAs.

For the subset analyses separating FGA and SGA exposures, we did not observe any SNP passing the genome-wide significance threshold (Table 2; Supplementary Figs. 3 & 5). There was also no evidence for population inflation for these two subsets with lambda values of 1.030 and 0.992 (Supplementary Figs. 4 & 6). The top suggestive SNPs from both subsets showed no excessive heterogeneity. The top affiliated genes from the FGA subset such as *NAV2*,* NRG3*, and *LSAMP* have been associated with autism and schizophrenia disorders (Must et al. 2008; Kao et al. 2010; Pretzsch and Ecker 2023). In addition, one of the top affiliated protein coding genes, *SHISA9* from the SGA subset has shown associations with epilepsy and autism disorder (Pfisterer et al. 2020).

Among overlapping SNPs at the 10^−03^ level, 1,463 (53%) SNPs in the SGA subset showed concordant effect directions with the FGA subset’s (*p* = 0.004), whereas 1,345 (48%) SNPs in the FGA subset aligned with the SGA subset’s SNPs (*p* = 0.995). The stronger directional agreement from SGA to FGA may reflect the broader pharmacological coverage of the FGA subset (which included mixed exposures). In contrast, the weaker concordance from FGA to SGA subset may suggest that top-associated SNP effects within the SGA subset may be more distinct given this subset included only exposure to SGA.

### EWAS results and permutation test results

In our primary within-case analysis including all EPSE samples, the most significant methylated position (DMP) associated with EPSE presence was cg05599348 (3.181 × 10^−07^), mapping to *TMSB15B* on chromosome X (hg19 position 103174718). This DMP was also one of the top identified DMPs in the FGA exposure subset (Table 3). However, this DMP was only present in the UCL samples while all other top identified DMPs were present in both samples. Thus, its overall signal may have been influenced by the relatively small sample size of the UCL samples. From the FGA/SGA exposure EWAS subsets, we also did not identify any DMP passing the threshold at 1 × 10^−07^ (Table 3). The top results from these three within-case design analyses involved mixed positive and negative methylation differences which reflect the complex epigenetic landscape associated the EPSE presence, likely involving multiple gene-specific regulatory changes.

**Table 3:** EPSE-associated differentially methylated positions

Probe ID	CHR	Position	Methylation difference (%)	SE (%)	*P* Value	Gene Annotation
EPSEs with exposure to any antipsychotic						
cg05599348	X	103,174,718	−5.439	0.980	3.181 × 10^−07^	*TMSB15B*
cg07679219	12	77,417,738	6.742	1.462	3.998 × 10^−06^	*E2F7*
cg06484572	6	41,605,494	1.686	0.369	4.769 × 10^−06^	*MDFI*
cg25194055	17	8,125,180	0.527	0.116	5.690 × 10^−06^	
cg00145438	13	113,105,097	−0.991	0.220	6.620 × 10^−06^	
cg26912312	20	61,274,254	−0.452	0.101	6.971 × 10^−06^	*SLCO4A1*
EPSEs with exposure to any type of FGA*						
cg00500167	6	100,841,663	0.464	0.098	2.093 × 10^−06^	*SIM1*
cg19185544	8	22,595,422	−1.748	0.371	2.418 × 10^−06^	*PEBP4*
cg05599348	X	103,174,718	−5.340	1.156	3.854 × 10^−06^	*TMSB15B*
cg05450477	6	20,426,845	1.654	0.369	7.293 × 10^−06^	*E2F3*
cg26875877	2	133,346,858	−0.040	0.886	7.742 × 10^−06^	*GPR39*
cg25030888	1	67,156,909	−3.013	0.692	1.327 × 10^−05^	*SGIP1*
EPSEs with exposure to SGA only						
cg11411904	1	153,935,719	1.051	0.215	3.341 × 10^−06^	*SLC39A1*
cg02388709	3	4,910,253	0.959	0.196	3.563 × 10^−06^	
cg21130374	21	42,734,266	−1.636	0.344	5.928 × 10^−06^	*MX2*
cg15977096	8	34,857,831	−4.410	0.965	1.268 × 10^−05^	
cg09583379	16	19,133,877	−6.745	1.479	1.316 × 10^−05^	
cg23814365	13	36,429,936	1.979	0.436	1.419 × 10^−05^	*DCLK1*
EPSEs with exposure to any antipsychotic compared to healthy controls						
cg14531564	1	1,154,853	2.949	0.423	3.073 × 10^−12^	***SDF4***
cg20647656	2	241,439,612	−1.413	0.236	2.098 × 10^−09^	***ANKMY1***
cg12524168	5	76,028,910	1.931	0.326	3.207 × 10^−09^	***F2R***
cg12004641	2	218,750,749	1.784	0.320	2.377 × 10^−08^	***TNS1***
cg05419385	12	27,352,945	1.565	0.281	2.549 × 10^−08^	
cg22583147	3	44,331,824	2.055	0.374	3.828 × 10^−08^	***TOPAZ1***
cg22845912	8	134,059,874	1.861	0.341	4.858 × 10^−08^	***SLA***
cg20730966	3	33,095,886	1.736	0.323	7.442 × 10^−08^	***GLB1***
cg12044923	4	5,207,312	1.695	0.316	8.414 × 10^−08^	***STK32B***
* Includes participants who had also been exposed to SGAs.						

*Notes.* Listed are all differentially methylated positions (DMPs) associated with different sets of EPSE samples. Sample sizes were: (1) 314 (UCL 64 EPSE cases, 33 EPSE controls; Aberdeen 47 EPSE cases, 170 EPSE controls); (2) 174 (UCL EPSE 57 cases, 23 EPSE controls; Aberdeen EPSE 30 cases, 64 controls); (3) 123 (UCL only 17 samples in total thus excluded; Aberdeen 17 EPSE cases, 106 EPSE controls); and (4) 859 (UCL 64 EPSE cases, 315 Healthy controls; Aberdeen 47 cases, 433 healthy controls). Results came from fixed model meta-analysis adjusted for participants’ methylation age, sex, and cell compositions. The comparison between EPSE cases and healthy controls adjusted for schizophrenia polygenic risk scores in addition to alleviate schizophrenia genetic risks. Positions are in hg19. Genes in bold had p meeting threshold at 1× 10^−07^.

* Includes participants who had also been exposed to SGAs.

Comparing EPSE cases with Healthy controls, we identified 9 DMPs associated with EPSE presence after controlling for schizophrenia PRS in addition (Table 3). Five of these identified DMPs have been implicated by past schizophrenia EWAS meta-analysis, cg12524168, *p* = 7.61 × 10^−20^; cg05419385, *p* = 3.08 × 10^−18^; cg22583147, *p* = 5.66 × 10^−22^; cg12044923, *p* = 1.32 × 10^−19^; and cg20730966, *p* = 4.90 × 10^−24^ (Hannon et al. 2016). The other four DMPs cg14531564, cg20647656, cg12004641, cg22845912, and their affiliated genes *SDF4*, *ANKMY1*, *TNS1*, *SLA* were not identified in past schizophrenia or smoking EWAS (Zeilinger et al. 2013; Elliott et al. 2014). Notably, most of the top DMPs had positive beta values except cg20647656. This trend toward hypermethylation may suggest that gene downregulation through increased methylation is a more prominent feature of EPSE.

We next examined whether the locations of these 9 DMPs could map to the corresponding GWAS of the same samples or to previously published schizophrenia GWAS. The GWAS summary statistics were first clumped so that multiple non-independent associations were collapsed into single associated loci. None of the identified DMPs were found to be associated with any genome-wide significant loci from past schizophrenia GWAS according to our regional mapping (Supplementary Fig. 7–15) (Trubetskoy et al. 2022). The SNP rs7622757 within a 250 kb window with cg22583147 was closest to genome-wide significance at *p* = 4.44 × 10^−07^ (Supplementary Fig. 12).

Our mapping of the DMPs to the GWAS of associated samples found that cg12044923 was significantly associated (permutation *p* = 0.010) with index SNP rs13108591 which had a GWAS p value of 7.482 × 10^−05^. cg20647656 was associated (permutation *p* = 0.030) with index SNP rs75037293 which had a GWAS p value of 2.73 × 10^−04^. According to the past schizophrenia GWAS (Trubetskoy et al. 2022), the SNPs rs13108591 (T/C) had p value of 0.761 and rs75037293 (G/C) had p value of 0.117 indicating minor relevance to schizophrenia (Trubetskoy et al. 2022). The SNP rs13108591 is located on chr4:5162317 (hg19), mapping to the intron of *STK32B*. SNP rs75037293 is located on chr2:241453995 (hg19) mapping to the intron of *ANKMY1*.

### PRS results

We found no evidence to suggest that the selected PRS could predict the development of EPSE (Table 4). According to the fixed model meta-analysis, genetic risk for schizophrenia (*p* = 0.566), Parkinson’s disease (*p* = 0.492), and Lewy-body dementia (*p* = 0.765) were not associated with the presence of EPSE.

**Table 4:** Results of multiple PRS regression analyses

Variables	Estimated Coefficient	Standard Error	Confidence Intervals (95%)	Reference Values	*p*
Schizophrenia PRS					
UCL	0.013	0.017	−0.020, 0.047	0.780	0.436
Aberdeen	0.006	0.020	−0.033, 0.046	0.321	0.749
UKB	0.032	0.017	−0.036, 0.032	1.941	0.053
Fixed-effect model	0.019	0.010	−0.001, 0.039	1.828	0.566
Parkinson’s disease PRS					
UCL	0.015	0.017	−0.018, 0.049	0.892	0.373
Aberdeen	0.025	0.022	−0.017, 0.068	1.162	0.246
UKB	−0.010	0.016	−0.042, 0.022	−0.637	0.525
Fixed-effect model	0.007	0.010	−0.013, 0.027	0.687	0.492
Lewy-body dementia PRS					
UCL	−0.022	0.016	−0.053, 0.009	−1.390	0.165
Aberdeen	0.017	0.023	−0.029, 0.062	0.718	0.473
UKB	0.026	0.017	−0.008, 0.060	1.481	0.139
Fixed-effect model	0.003	0.011	−0.017, 0.024	0.299	0.765

*Notes.* *PRS* polygenic risk scores

All results were adjusted for participants’ age, sex, genotyping chip-type, and the first three principal components from GWAS population stratification. Reference values were t values for individual models and z values for the fixed effect model.

## Discussion

In the present study, we report the largest GWAS meta-analysis of EPSE and the first EWAS meta-analysis of EPSE in European populations to date with exploration on the effects of FGA and SGA exposure. The prevalence of any type of EPSE was found to be 48% among participants who have taken either FGA or SGA. EPSEs were found to be more prevalent among those who had taken FGA or both. No SNP passed the genome-wide threshold of significance. The top index SNP rs2709733 from the GWAS of all antipsychotic exposure mapped to a long intergenic non-protein coding RNA, *LINC01162* with consistent effect across all cohorts. SNPs associated with EPSEs from exposure to FGAs at the suggestive level mapped to *NAV2*,* NRG3*, and *LSAMP*. SNPs associated with EPSEs from exposure to SGA mapped to *SHISA9* and *CNBD1*. The primary EWAS meta-analysis indicated suggestive gene *TMSB15*B, located on chromosome X. In addition, we identified multiple DMPs associated with EPSE passing the significance threshold comparing EPSE cases to healthy controls. The *STK32B* gene which was implicated by methylation probe cg12044923 has been associated with psychiatric and movement disorders. We found no evidence that PRSs for schizophrenia, Parkinson’s, and Lewy-body dementia predict EPSE development.

The GWAS meta-analysis results may represent a false negative due to the limited sample size and power. Other factors may also be relevant. For example, our sign tests revealed weak SNP effect alignment between the FGA and the SGA GWAS results, suggesting that there may be differences in the genetic architecture of these traits. Thus, combining participants who have taken either FGA or SGA may have impacted our ability to identify drug-specific genetic risks. Conversely, stratifying the sample by antipsychotic exposure (FGA vs. SGA) substantially reduced sample sizes, compromising statistical power. However, several of the implicated genes (*NAV2*,* NRG3*,* LSAMP* and *SHISA9*) from the subset analyses were previously reported to be associated with psychiatric disorders and neuronal functions (Must et al. 2008; Kao et al. 2010; Pfisterer et al. 2020; Pretzsch and Ecker 2023). These findings warrant cautious interpretation due to the suggestive nature of the associations. The results produced here are the result of a concerted effort to increase sample size as a starting point for future studies.

The limited GWAS and PRS findings lead us to speculate that EPSE may be more strongly driven by epigenetic modifications over time. Studies have suggested that methylation changes in dopaminergic or serotonergic pathway genes may impact motor control pathways more dynamically than SNP-based variations (Loke et al. 2015). This dynamic epigenetic regulation aligns with how EPSE can vary significantly among patients and change with continued antipsychotic use, whereas GWAS-derived SNPs only offer a static view of genetic risk. Therefore, integrating EWAS may provide insights into the gene-environment interactions involved in EPSE development.

Our primary EWAS analysis of EPSE status may have been again limited by modest sample size, potentially reducing statistical power to detect robust epigenetic associations. However, permutation testing in our expanded EWAS comparing EPSE cases with healthy controls identified two genes, *ANKMY1* and *STK32B*, showing significant relevance to the presence of EPSE. *ANKMY1* encodes the protein Ankyrin Repeat and MYND Domain Containing 1, which has a role for protein-protein interactions and cellular signalling. This could indirectly influence pathways relevant to neurodevelopment or dopamine signalling. However, we have found little additional corroborating evidence directly linking *ANKMY1* to schizophrenia or EPSE. The other implicated gene *STK32B* encodes for a member of the human N-myristoylated proteins, which are involved in various cellular signalling and transduction pathways, although its exact biological function remains insufficiently defined (Takamitsu et al. 2015). A 520-kb homozygous deletion encompassing *STK32B* has been described in Ellis-Van-Creveld syndrome, which is a rare genetic disorder that primarily affects the skeletal system and other tissues (Temtamy et al. 2008).

Notably, changes in the methylation of the *STK32B* promoter region have been previously linked to both schizophrenia and anxiety disorders. This protein may play a role in executive functions such as working memory and selective attention (Hannon et al. 2016; Ciuculete et al. 2018). Moreover, *STK32B* was implicated in a GWAS of essential tremor (Müller et al. 2016), and patients with essential tremor showed increased expression of *STK32B* in the cerebellar cortex, highlighting a potential relevance to movement abnormalities. The positive beta value observed for *STK32B* in the present study may suggest that its downregulation is associated movement side-effects. This increased methylation may come from the participants’ lifestyle factors or long-term exposure to antipsychotics. Future functional studies are needed to clarify the exact role of *STK32B* in EPSE development.

The current study has several limitations. First, the study’s EPSE classification was based on cross-sectional data from existing studies. Individuals classified as not having EPSE at the time of assessment may develop EPSE later in life with increasing exposure to antipsychotics, introducing potential pseudo-negatives. In addition, we could not differentiate between acute and chronic EPSE. Medication dose information was unavailable for most participants thus, not analysed. We used a mixed definition of EPSE and mixed antipsychotic medications and EPSE medications, each of which may have distinct profiles of EPSE risk. Other dynamic factors such as aging, comorbid conditions and drug-drug interactions may influence the recognition of EPSE as well. Differences in sample ascertainment may have contributed to variability in EPSE detection sensitivity and the predominance of male participants could be another source of bias. These variability and potential miss-classification could impact the consistency of our findings and warrant careful consideration in future studies to clarify the effects of specific antipsychotic medications on EPSE with increased sample size to do so. Finaly, although we have implemented strategies to control for collider bias related to schizophrenia, our results may still be influenced by genetic risk to schizophrenia.

Overall, our study provides new insights into the biological mechanisms underlying EPSE development in patients with schizophrenia. Notably, our approach integrated findings from EWAS with GWAS results, allowing us to explore EPSE-associated methylation shifts using accessible SNP data. The findings of this study indicate that further investigation of the epigenetics of EPSE and the role of *STK32B* in EPSE is likely to enhance our understanding and inform future research and treatment directions.

## Supplementary Information

Below is the link to the electronic supplementary material.

Supplementary Material 1(DOCX 121 bytes)

## Data Availability

The raw and processed data are available from the corresponding author upon request.
