# Supplementary material for: Integrating genome-wide and epigenome-wide associations for antipsychotic induced extrapyramidal side effects
Source: Psychopharmacology (Berl). 2025 Oct 11;243(7):1631–42. doi: 10.1007/s00213-025-06912-w (PMC13433426; doi:10.1007/s00213-025-06912-w)
Supplement: Supplementary file 1 [file 213_2025_6912_MOESM1_ESM.docx]

**Supplementary Materials**

**Integrating Genome-wide and Epigenome-wide Associations for Antipsychotic Induced Extrapyramidal Side Effects**

Kai Yao^1^, Johan H. Thygesen^2^, Siobhan K. Lock^3^, Antonio F. Pardiñas^3^, Antonia L. Pritchard^4^, Israa Elbashir^1^, Abdullah Lodhi^1^, Zain-Ul-Abideen Ahmad^1^, Michael C. O’Donovan^3^, Michael J. Owen^3^, James T. R. Walters^3^, David St Clair^5^, Nick Bass^1^, Andrew McQuillin^1^

^1^ Molecular Psychiatry Laboratory, Division of Psychiatry, University College London, Rockefeller Building, 21 University Street, London, WC1E 6BT, UK

^2^ Institute of Health Informatics, University College London, London, UK

^3^ Centre for Neuropsychiatric Genetics and Genomics, School of Medicine, Cardiff University, Hadyn Ellis Building, Cardiff, CF24 4HQ, UK

^4^ Division of Biomedical Science, University of the Highlands and Islands, An Lochran, Inverness, IV2 5NA, UK

^5^ School of Medicine, Medical Sciences and Nutrition University of Aberdeen, King's College, Aberdeen, AB24 3FX, UK

**Corresponding Author**

Andrew McQuillin

*Molecular Psychiatry Laboratory, Division of Psychiatry, University College London, Rockefeller Building, 21 University Street, London, WC1E 6BT, UK*

[a.mcquillin@ucl.ac.uk](mailto:a.mcquillin@ucl.ac.uk) +44 20 3108 2181

**Table of Contents**

[Supplementary Table 1. Behavioural Keywords for EPSE Selection	3](#_Toc202886386)

[Supplementary Table 2. Pharmacological Keywords for EPSE Selection	4](#_Toc202886387)

[Supplementary Table 3. First Generation Antipsychotic Codes for UKB Participants	5](#_Toc202886388)

[Supplementary Table 4. Second Generation Antipsychotic Codes for UKB Participants	6](#_Toc202886389)

[Supplementary Table 5. EPSE Medication Codes for UKB Participants	7](#_Toc202886390)

[Supplementary Table 6. GWAS Participants’ Characteristics concerning EPSE Presence	8](#_Toc202886391)

[Supplementary Table 7. The Distribution of EPSE identified from Different Key Words in UCL Samples	9](#_Toc202886392)

[Supplementary Figure 1. The Manhattan Plot of Primary EPSE GWAS Meta-analysis	10](#_Toc202886393)

[Supplementary Figure 2. The QQ Plot of Primary EPSE GWAS Meta-analysis	11](#_Toc202886394)

[Supplementary Figure 3. The Manhattan Plot of EPSE GWAS Meta-analysis for any Exposure to FGA	12](#_Toc202886395)

[Supplementary Figure 4. The QQ Plot of EPSE GWAS Meta-analysis for any Exposure to FGA	13](#_Toc202886396)

[Supplementary Figure 5. The Manhattan Plot of EPSE GWAS Meta-analysis for only Exposure to SGA	14](#_Toc202886397)

[Supplementary Figure 6. The QQ Plot of EPSE GWAS Meta-analysis for only Exposure to SGA	15](#_Toc202886398)

[Supplementary Table 8. EWAS Case-control Design Participants’ Demographics and Clinical Characteristics concerning EPSE Presence	17](#_Toc202886399)

[Supplementary Figures 7–15 Mappings of Significant CpG Sites to Schizophrenia GWAS within a 250kb window	18](#_Toc202886400)

| **Symptoms** | **Keywords** |
| --- | --- |
| General terms for EPSE | Extrapyramidal; extra pyramidal; epse; EPSE; movement disorder |
| Parkinsonism (tremor) | Tremor; tremble; shake; quiver |
| Parkinsonism (rigidity) | Rigid; hypertonic; stiff; inflexible; tight; tense; muscle pain |
| Parkinsonism (Sialorrhea) | Salivation; sialorrhea; drool |
| Parkinsonism (bradykinesia and hypokinesia) | Hypokinesia; bradykinesia; freeze; froze; slow; motor block; stride; reduced arm swing; blink; hypophonia; soft voice; voice volume; slurred; micrographia; handwriting |
| Parkinsonism (mask face) | Mask; hypomimia; expressionless |
| Parkinsonism (stooped posture) | Stoop; hunch; posture |
| Parkinsonism (parkinsonian gait) | Shuffle; festinate; parkinsonian |
| Parkinsonism (parkinsonian gait) | Imbalance; fall |
| Dystonia (general) | Dystonia; jerk; twist; twitch; spasm; lock |
| Dystonia (opisthotonos) | Opisthotonos; arch back; bend back; arch spine; bend spine |
| Dystonia (torticollis) | Torticollis; cervical dystonia; head deviation; head posturing; neck pain |
| Dystonia (oculogyric crisis) | Oculogyric; fix stare; fix eye; deviate eye |
| Dystonia (trismus) | Trismus; lock jaw; lockjaw; jawlock; jaw deviation; jaw retraction; clench; grind; mouth pain; restrict mouth; limit mouth |
| Dystonia (tortipelvic crisis) | Tortipelivic; bend trunk; twist trunk |
| Dystonia (buccolingual crisis) | Buccolingual; dysphagia; difficult swallow; grimace; protrude; protrusion; risus sardonicus; dysarthria; difficult speak; difficult speech; pseudomacroglossia; swollen tongue; tongue swell |
| Dystonia (laryngeal dystonia) | Laryngeal; stridor; strangled voice; breathy voice; quiet voice; whispery voice; hoarse; shaky voice; aphonia; interrupt speech; lose voice; voice loss |
| Tardive Dyskinesia (general) | tardive dyskinesia; dyskinesia; TD |
| Tardive Dyskinesia (orofacial dyskinesia) | Involuntary; tongue twist; tongue protrusion; chew; biting; bite; suck; clench; lateral jaw movement; sideway jaw movement; smack lip; lip purse; pucker; puff cheek; frown; blink; grimace; blink |
| Tardive Dyskinesia (limb truncal dyskinesia) | Choreiform; athetoid; choreoathetoid; purposeless; rock; twist; squirm; gyrate; thrust; knee move; tap; heel drop; writhing; rotate; nod; inversion; eversion |
| Akathisia | Akathisia; akathisia; restless; pace; fidget; irritable; leg cross; leg swing; foot shift; shuffle; tramp; still; march; shift weight; rock |

**Supplementary Table 1. Behavioural Keywords for EPSE Selection**

**Supplementary Table 2. Pharmacological Keywords for EPSE Selection**

| **Medication** | **Keywords** |
| --- | --- |
| Trihexyphenidyl | Trihex; benzh; artane; agitane; parkin |
| Benzatropine | Benza;tropin; cogentin |
| Procyclidine | Procy; lidin; kemad |
| Orphenadrine | Orphan; drine |
| Biperiden | Biper; akineton |
| Hyoscine | Hyos; kwell |
| Tetrabenazine | Tetrab; ranb; nitom; xenaz |

**Supplementary Table 3. First Generation Antipsychotic Codes for UKB Participants**

| **Medication Name** | **Medication Type** | **Medication Code** |
| --- | --- | --- |
| Benperidol | anquil 250micrograms tablet; benperidol | 1140867080; 1140867078 |
| Chlorpromazine | chlorpromazine; cpz - chlorpromazine; largactil 10mg tablet; chloractil 25mg tablet | 1140879658; 1140910358; 1140863416; 1140863410 |
| Flupentixol | flupentixol; depixol 3mg tablet; fluanxol 500micrograms tablet; flupenthixol; flupentixol | 1140909800; 1140867152; 1140867952; 1140867150; 1140909800 |
| Fluphenazine | decazate 25mg/1ml oily injection; fluphenazine; fluphenazine decanoate; modecate 12.5mg/0.5ml oily injection; moditen 1mg tablet; moditen enanthate 25mg/ml injection | 1140867474; 1140882098; 1140867398; 1140867456; 1140867156; 1140856004 |
| Haloperidol | haldol 5mg tablet; haloperidol; serenace 500micrograms capsule | 1140867184; 1140867168; 1140867092 |
| Levomepromazine | levomepromazine; nozinan 25mg tablet | 1140909802; 1140867122 |
| Loxapine | loxapine; loxapac 10mg capsule | 1140867406; 1140867414 |
| Pericyazine | neulactil 2.5mg tablet; pericyazine | 1140867136; 1140867134 |
| Perphenazine | fentazin 2mg tablet; perphenazine | 1140867210; 1140867208 |
| Pimozide | orap 2mg tablet; pimozide | 1140867272; 1140867218 |
| Pipotiazine | piportil depot 50mg/1ml oily injection; pipotiazine | 1140867572; 1140909804 |
| Prochlorperazine | prochlorperazine; stemetil 5mg tablet | 1140868170; 1140868172 |
| Promazine | promazine | 1140879746 |
| Sulpiride | dolmatil 200mg tablet; sulparex 200mg tablet; sulpiride; sulpitil 200mg tablet; sulpor 200mg/5ml oral solution | 1140867306; 1140917366; 1140867304; 1140882376; 1141185130 |
| Thioridazine | thioridazine; melleril 10mg tablet | 1140879750; 1140867312 |
| Trifluoperazine | stelazine 1mg tablet; tranylcypromine+trifluoperazine 10mg/1mg tablet; trifluoperazine | 1140867244; 1140867944; 1140868120 |
| Zuclopenthixol | clopixol 2mg tablet; zuclopenthixol | 1140867342; 1140882100 |

**Supplementary Table 4. Second Generation Antipsychotic Codes for UKB Participants**

| **Medication Name** | **Medication Type** | **Medication Code** |
| --- | --- | --- |
| Amisulpride | amisulpride; solian 100mg/ml s/f oral solution | 1141153490; 1141184742 |
| Aripiprazole | abilify 5mg tablet; aripiprazole | 1141202024; 1141195974 |
| Clozapine | clozapine; clozaril 25mg tablet; denzapine 25mg tablet | 1140867420; 1140882320; 1141200458 |
| Olanzapine | olanzapine; zyprexa 2.5mg tablet | 1140928916; 1141167976 |
| Oxypertine | oxypertine; integrin 10mg capsule | 1140879754; 140855978 |
| Quetiapine | quetiapine; seroquel 25mg tablet | 1141152848; 1141152860 |
| Remoxipride | remoxipride; roxiam 150mg m/r capsule | 1140879704; 1140867432 |
| Risperidone | dozic 1mg/ml oral liquid; risperdal 0.5mg tablet; risperidone | 1140867180; 1141177762; 1140867444 |
| Sertindole | serdolect 4mg tablet; sertindole | 1140927970; 1140927956 |

| **Medication Name** | **Medication Type** | **Medication Code** |
| --- | --- | --- |
| Akineton | akineton | 1140872522 |
| Artane | artane | 1140872378 |
| Benzatropine | benzatropine | 1140909818 |
| Benzhexol | benzhexol | 1140883510 |
| Biperiden | biperiden | 1140872520 |
| Cogentin | cogentin | 1140872460 |
| Kemadrin | kemadrin | 1140872542 |
| Orphenadrine | orphenadrine | 1140883560 |
| Procyclidine | procyclidine | 1140883476 |
| Tetrabenazine | tetrabenazine | 1140872556 |
| Tetrabenazine Product | tetrabenazine_product | 1141157336 |
| Trihexyphenidyl | trihexyphenidyl | 1140909816 |
| Xenazine | xenazine | 1141171726 |

**Supplementary Table 5. EPSE Medication Codes for UKB Participants**

|  |  | **N** | **Overall** | **EPSE Presence** | **EPSE Absence** | **Neff** | **p-values** |
| --- | --- | --- | --- | --- | --- | --- | --- |
| **UCL** |  | *1017* |  | *n = 587 (58%)* | *n = 430 (42%)* | *983* |  |
|  | **Age at assessment** | 1017 | 44.80 (12.24) | 46.33 (12.10) | 42.72 (12.14) |  | **<0.001**^a^ |
|  | **Age of onset** | 761 | 23.34 (8.18) | 23.28 (8.07) | 23.44 (8.34) |  | 0.795^a^ |
|  | **Sex** | 1017 |  |  |  |  | 0.145^b^ |
|  | Male |  | 735 (72%) | 435 (74%) | 300 (70%) |  |  |
|  | Female |  | 282 (28%) | 152 (26%) | 130 (30%) |  |  |
|  | **Antipsychotics** | 973 |  | |  |  | **<0.001**^b^ |
|  | First generation |  | 102 (10%) | 77 (14%) | 25 (6%) |  |  |
|  | Second generation |  | 452 (47%) | 208 (37%) | 244 (59%) |  |  |
|  | Both generations |  | 419 (43%) | 274 (49%) | 145 (35%) |  |  |
| **Aberdeen** | | *414* |  | *n = 90 (22%)* | *n = 324 (78%)* | *282* |  |
|  | **Age at assessment** | 414 | 44.40 (13.20) | 45.70 (13.12) | 44.03 (13.21) |  | 0.292^a^ |
|  | **Age of onset** | 401 | 24.08 (8.05) | 23.27 (7.76) | 24.29 (8.13) |  | 0.293^a^ |
|  | **Sex** | 414 |  |  |  |  | 0.426^b^ |
|  | Male |  | 311 (75%) | 71 (79%) | 240 (74%) |  |  |
|  | Female |  | 103 (25%) | 19 (21%) | 84 (26%) |  |  |
|  | **Antipsychotics** | 414 |  | |  |  | **<0.001**^b^ |
|  | First generation |  | 135 (33%) | 42 (47%) | 93 (29%) |  |  |
|  | Second generation |  | 231 (56%) | 32 (35%) | 199 (61%) |  |  |
|  | Both generations |  | 48 (11%) | 16 (18%) | 32 (10%) |  |  |
| **UKB** |  | *507* |  | *n = 90 (18%)* | *n = 417 (82%)* | *296* |  |
|  | **Age at assessment** | 507 | 54.32 (8.08) | 56.06 (7.61) | 53.94 (8.14) |  | **0.020**^a^ |
|  | **Sex** | 507 |  |  |  |  | 0.322^b^ |
|  | Male |  | 335 (66%) | 64 (71%) | 271 (65%) |  |  |
|  | Female |  | 172 (34%) | 26 (29%) | 146 (35%) |  |  |
|  | **Antipsychotics** | 507 |  | |  |  | **<0.001**^b^ |
|  | First generation |  | 158 (31%) | 52 (58%) | 106 (25%) |  |  |
|  | Second generation |  | 320 (63%) | 28 (31%) | 292 (70%) |  |  |
|  | Both generations |  | 29 (6%) | 10 (11%) | 19 (5%) |  |  |
| **Cardiff** | | *533* |  | *n = 411 (77%)* | *n = 122 (23%)* | *376* |  |
|  | **Age at assessment** | 533 | 44.30 (11.70) | 44.90 (10.90) | 42.20 (14.10) |  | 0.057^a^ |
|  | **Age of onset** | 508 | 25.00 (8.80) | 24.30 (7.60) | 27.50 (11.60) |  | **0.006**^a^ |
|  | **Sex** | 533 |  |  |  |  | 0.900^b^ |
|  | Male |  | 346 (65%) | 268 (65%) | 78 (64%) |  |  |
|  | Female |  | 187 (35%) | 143 (35%) | 44 (36%) |  |  |
|  | **Antipsychotics** | 531 |  | |  |  | 0.100^b^ |
|  | First generation |  | 93 (17%) | 78 (19%) | 15 (12%) |  |  |
|  | Second generation |  | 433 (82%) | 327 (80%) | 106 (88%) |  |  |
|  | Both generations |  | 5 (1%) | 5 (1%) | 0 (0%) |  |  |
| *Notes.* EPSE, extrapyramidal side effects; SD, standard deviation, In bold p passed significance threshold  ^a^ Two Simple t-test; mean (SD); ^b^ Pearson's Chi-squared test of independence; n (%)  Neff, effective sample sizes, calculated as 4/(1/n_cases + n_controls); UCL Neff is a sum of Neff from 3 separate genotyping waves (Affymetrix 125; PsychArray 579; Global Screening Array 279) | | | | | | | |

**Supplementary Table 6. GWAS Participants’ Characteristics concerning EPSE Presence**

**Supplementary Table 7. The Distribution of EPSE Coding identified from Different Key Words in UCL Samples**

|  | **Total** | **FGA Subgroup** | **SGA Subgroup** |
| --- | --- | --- | --- |
|  | *559* | *351* | *208* |
| **General EPSE** | 59 (11%) | 53 (15%) | 6 (3%) |
| **Dyskinesia** | 32 (6%) | 26 (7%) | 6 (3%) |
| **Parkinsonism** | 327 (58%) | 196 (56%) | 131 (63%) |
| **Akathisia** | 91 (16%) | 62 (18%) | 29 (14%) |
| **Dystonia** | 59 (11%) | 41 (12%) | 18 (9%) |
| **EPSE Medication** | 183 (33%) | 120 (34%) | 63 (30%) |
| **More than one presentation** | 175 (31%) | 123 (35%) | 52 (25%) |

*Notes.* FGA, first generation antipsychotics; SGA, second generation antipsychotics.

FGA subgroup contained samples with any exposure to FGA including SGA exposure. SGA subgroup contained samples with only exposure to SGA. The coding separation reflects key words in supplementary tables 1 and 2.

**Supplementary Figure 1. The Manhattan Plot of Primary EPSE GWAS Meta-analysis**

**Supplementary Figure 2. The QQ Plot of Primary EPSE GWAS Meta-analysis**

*Notes.* X-axis (Expected -log10(p-values)): Represents the theoretical quantiles under the null hypothesis, where p-values are uniformly distributed on a logarithmic scale; Y-axis (Observed -log10(p-values)): Shows the observed -log10(p-values) from the GWAS.

**Supplementary Figure 3. The Manhattan Plot of EPSE GWAS Meta-analysis for any Exposure to FGA**

**Supplementary Figure 4. The QQ Plot of EPSE GWAS Meta-analysis for any Exposure to FGA**

*Notes.* X-axis (Expected -log10(p-values)): Represents the theoretical quantiles under the null hypothesis, where p-values are uniformly distributed on a logarithmic scale; Y-axis (Observed -log10(p-values)): Shows the observed -log10(p-values) from the GWAS.

**Supplementary Figure 5. The Manhattan Plot of EPSE GWAS Meta-analysis for only Exposure to SGA**

**Supplementary Figure 6. The QQ Plot of EPSE GWAS Meta-analysis for only Exposure to SGA**

*Notes.* X-axis (Expected -log10(p-values)): Represents the theoretical quantiles under the null hypothesis, where p-values are uniformly distributed on a logarithmic scale; Y-axis (Observed -log10(p-values)): Shows the observed -log10(p-values) from the GWAS.

*EWAS Case-control Design Sample Demographics*

The UCL participants who developed EPSE were younger than the healthy controls in terms of age at assessment (36.90 vs 44.48, *p*<0.001; See Supplementary Table S7) and methylation age (39.54 vs 44.08, *p*=0.008). The UCL EPSE cases also had higher ratios of males (81% vs 44%, *p*<0.001). The Aberdeen participants’ methylation age (54.29 vs 53.04, *p*=0.473), and males’ ratio (70% vs 74%, *p*=0.737) were balanced between the EPSE and the control group.

|  |  | **N** | **Overall** | **EPSE Presence** | **Controls** | **p-values** |
| --- | --- | --- | --- | --- | --- | --- |
| **UCL** |  | 379 |  | n = 64 (17%) | n = 315 (83%) |  |
|  | **Age at assessment** | 362 | 38.24 (14.81) | 36.90 (14.74) | 44.48 (13.58) | **<0.001**^a^ |
|  | **mAge (Horvath)** | 379 | 40.31 (12.46) | 39.54 (12.41) | 44.08 (12.13) | **0.008**^a^ |
|  | **Sex** |  |  |  |  | **<0.001**^b^ |
|  | Male |  | 192 (51%) | 52 (81%) | 140 (44%) |  |
|  | Female |  | 187 (49%) | 12 (19%) | 175 (56%) |  |
|  | **Antipsychotics** | 64 |  |  |  |  |
|  | First generation |  |  | 14 (22%) | / |  |
|  | Second generation |  |  | 6 (9%) | / |  |
|  | Both Generations |  |  | 44 (69%) | / |  |
| **Aberdeen** | | 480 |  | n = 47 (10%) | n = 433 (90%) |  |
|  | **mAge (Horvath)** | 480 | 53.16 (9.88) | 54.29 (11.48) | 53.04 (9.70) | 0.473^a^ |
|  | **Sex** | 480 |  |  |  |  |
|  | Male |  | 352 (73%) | 33 (70%) | 319 (74%) | 0.737^b^ |
|  | Female |  | 128 (27%) | 14 (30%) | 114 (36%) |  |
|  | **Antipsychotics** | 47 |  |  |  |  |
|  | First generation |  |  | 24 (51%) | / |  |
|  | Second generation |  |  | 17 (36%) | / |  |
|  | Both generations |  |  | 6 (13%) | / |  |
| *Notes.* EPSE, extrapyramidal side effects; SD, standard deviation  ^a^ Two Simple t-test; mean (SD)  ^b^ Pearson's Chi-squared test of independence; n (%)  In bold p passed significance threshold | | | | | | |

**Supplementary Table 8. EWAS Case-control Design Participants’ Demographics and Clinical Characteristics concerning EPSE Presence**

**Supplementary Figures 7–15 Mappings of Significant CpG Sites to Schizophrenia GWAS within a 250kb window**

Black line in the central mark the SNP closest to the CpG. Red dotted line marked the SNP in lead within the region and other nearby SNPs in linkage disequilibrium.

**Supplementary Figure 7. The Mapping of CpG cg14531564 to the Schizophrenia GWAS Region**

**Supplementary Figure 8. The Mapping of CpG cg20647656 to the Schizophrenia GWAS Region**

**Supplementary Figure 9. The Mapping of CpG cg12524168 to the Schizophrenia GWAS Region**

**Supplementary Figure 10. The Mapping of CpG cg12004641 to the Schizophrenia GWAS Region**

**Supplementary Figure 11. The Mapping of CpG cg05419385 to the Schizophrenia GWAS Region**

**Supplementary Figure 12. The Mapping of CpG cg22583147 to the Schizophrenia GWAS Region**

**Supplementary Figure 13. The Mapping of CpG cg22845912 to the Schizophrenia GWAS Region**

**Supplementary Figure 14. The Mapping of CpG cg20730966 to the Schizophrenia GWAS Region**

**Supplementary Figure 15. The Mapping of CpG cg12044923to the Schizophrenia GWAS Region**
